# *BCAbox* Algorithm Expands Capabilities of Raman Microscope for Single Organelles Assessment

**DOI:** 10.3390/bios8040106

**Published:** 2018-11-10

**Authors:** Andrey N. Kuzmin, Artem Pliss, Alex Rzhevskii, Adrian Lita, Mioara Larion

**Affiliations:** 1 Advanced Cytometry Instrumentation Systems, LLC, Buffalo, NY 14203, USA; lptacis@gmail.com or ampliss@buffalo.edu; 2 Institute for Lasers, Photonics and Biophotonics, University at Buffalo, State University of New York, Buffalo, NY 14260, USA; 3 Thermo Fisher Scientific, Waltham, MA 02451, USA; alexander.rzhevskii@thermofisher.com; 4 Neuro-Oncology Branch, Center for Cancer Research, National Cancer Institute, Bethesda, MD 20892, USA; adrian.lita@nih.gov (A.L.); mioara.larion@nih.gov (M.L.)

**Keywords:** Raman spectrometry, microscopy, biomolecular analysis, single cell assay

## Abstract

Raman microspectroscopy is a rapidly developing technique, which has an unparalleled potential for in situ proteomics, lipidomics, and metabolomics, due to its remarkable capability to analyze the molecular composition of live cells and single cellular organelles. However, the scope of Raman spectroscopy for bio-applications is limited by a lack of software tools for express-analysis of biomolecular composition based on Raman spectra. In this study, we have developed the first software toolbox for immediate analysis of intracellular Raman spectra using a powerful biomolecular component analysis (BCA) algorithm. Our software could be easily integrated with commercial Raman spectroscopy instrumentation, and serve for precise analysis of molecular content in major cellular organelles, including nucleoli, endoplasmic reticulum, Golgi apparatus, and mitochondria of either live or fixed cells. The proposed software may be applied in broad directions of cell science, and serve for further advancement and standardization of Raman spectroscopy.

## 1. Introduction

Raman spectroscopy and microscopy, in particular, holds its unique niche in the ever-expanding family of biosensing tools, bridging conventional microscopic imaging together with a molecular analysis of the samples. This optical technique utilizes inelastic light scattering from the molecular bond vibrations, which produce their characteristic Raman spectral bands, enabling selective detection of diverse biomolecules in a sample that includes certain amino acids, DNA, and RNA, as well as various classes of lipids, proteins, and saccharides. Furthermore, Raman spectroscopy does not require any staining, it does not destroy the samples, and could be applied for studies of live cells and tissues. In addition, the intensity of a Raman band of a specific constituent is directly proportional to its concentration, thus enabling quantitative analysis, which can be performed in microdomains [1,2] of a cell. For all these benefits, Raman spectroscopy has been increasingly utilized in proteomics [3,4], transcriptomics [5], and metabolomics research [6,7]. In fact, maturation of the Raman spectroscopic technology leads to an emergence of Ramanomics, an independent “Omics” discipline, aimed at quantitative characterization and monitoring of molecular content in biological samples [8].

A growing number of studies have applied Raman microscopy for molecular analysis of compartmentalized structure in eukaryotic cells. Since the waist of the focused laser beam in a confocal Raman microscope is smaller than most cellular organelles, it could be scanned over a cell to acquire Raman spectra in distinct subcellular structures, to probe the local molecular environment. Recently emerged single-organelle spectroscopy has already proven itself as an invaluable approach for characterization of cellular macromolecular structures [9,10,11,12,13], identification and monitoring of cellular heterogeneity [14], cellular differentiation [15], drug-cell interactions [16], and detection of biomarkers of diseases, such as cancer [17]. However, the quantitative analysis of Raman spectra has not been sufficiently developed yet. Among different bioanalytical approaches for the processing of Raman spectra, biomolecular component analysis (BCA) [8,18] is arguably the most informative, as it identifies the concentrations of specific molecular groups into the sites of acquisition. BCA is based on an accurate spectral fit of a model spectrum, generated by the linear summation of the weighted spectra of the basic biomolecular components, into a measured Raman spectrum of biological samples. The spectral weights, which are optimized during the fitting procedure, directly yield the concentrations of specific types of biomolecules. BCA measurements in subcellular structures have been consistent with values obtained by other bioanalytical techniques, including interference microscopy [19], fluorescence lifetime imaging [20], and quantitative cryo-electron microscopy [21]. While the groundbreaking potential of confocal Raman micro-spectroscopy—BCA has been already validated in diverse biomedical applications [8,10,13,14,15,16,17,22,23,24], BCA for a commercial Raman microscope tool has yet to be implemented. Presently, BCA algorithms are developed by the end-users in Matlab or similar software environments, while to our knowledge, there is no commercially available Raman microspectrometer with an embedded BCA toolbox for multi-cycle measurements of biomolecular concentrations in the samples.

Here we present and discuss an implementation of stand-alone *BCAbox* software (ACIS, LLC, Buffalo, NY, USA) on the basis of a commercial confocal Raman microscope. The major advantages of this software are that it is intended for biomolecular component analysis of cellular organelles in-situ, and that it can determine the absolute concentrations if hardware is calibrated accordingly. Up-to-date the beta version of *BCAbox* is the only commercial software, which supports MicroRaman-BCA.

The BCA toolbox presented here was optimized and validated for molecular profiling of several types of specific organelles: nucleoli, endoplasmic reticulum, Golgi apparatus, and mitochondria both in live and fixed cells. Obtained protocols and software will help to standardize the Raman spectroscopy applications and make it available for researchers with different levels of prior training. The BCA toolbox is aimed at molecular profiling of single cellular organelles and is suitable for a broad range of biomedical assays.

## 2. Materials and Methods

Raman instrumentation. A DXR2 Raman confocal microscope (Thermo Fisher Scientific, Waltham, MA, USA) was equipped with a 633 nm @70 mW Laser Diode module ROUSB-633-PLR-70-1 (Ondax, Monrovia, CA, USA) to produce ~33 mW of excitation power at the sample. A Fluorescence Illuminator with a green fluorescence Cube (510–550 nm) and an X-Cyte^TM^ 120-PC (Photonic Solutions Inc., Mississauga, ON, Canada) mercury lamp were installed for visualization of labelled cellular organelles. The high resolution blazing-angle (633 nm) grating (50–1900 cm^−1^ spectral range, 2 cm^−1^ full width at half maximum) and Plan N 100× (NA = 1.25, Olympus, Waltham, MA, USA) oil immersion objective were chosen for Raman spectra acquisition to be compatible with BCA.

Measurement reproducibility. To determine standard deviations in spectral measurements, Raman spectra of freshly extracted egg whites were measured at the same accumulation times as in the experiments with cultured cells. Protein concentration in egg whites is close to that of intracellular proteins, and thus mimics a homogeneous biomolecular medium. The standard deviations of the measured spectra were found to be below 5%, which warrants reproducibility of measurements (see Figure S1).

Subtraction of the Raman background. An internal algorithm in the BCA toolbox for an automatic subtraction of background from the spectra acquired in the cells growing on a glass dish bottom was developed (Figure S2). To ensure the accuracy of this procedure, all background components for glass-bottom Petri dishes were measured, smoothed, and normalized to be used by software for background subtraction (Figure S3).

Instrument calibration. The Raman microscope was calibrated for measurements of absolute concentrations of proteins, DNA, RNA, lipids, and saccharides, using aqueous solutions of bovine serum albumin (Sigma-Aldrich, St. Louis, MO, USA), calf thymus DNA (Sigma-Aldrich, St. Louis, MO, USA), S. cerevisiae RNA (Sigma-Aldrich, St. Louis, MO, USA), and Type IX glycogen from bovine liver (Sigma-Aldrich, St. Louis, MO, USA). Additionally, a chloroform solution of bovine brain lipids (Avanti Polar Lipids, Inc., Alabaster, AL, USA) was used. To avoid fast evaporation, measurements of the lipid solution in chloroform were done in sealed capillaries. Raman profiles of the BCA components for initial modeling were measured using DNA and RNA extracts obtained from HeLa cells for DNA and RNA components, respectively. The lipid component was measured in lipid droplets of HeLa live cells. Initial organellar protein profiles were obtained by subtraction of all other weighted BCA components. Then, as measured data was accumulated, profiles of RNA, DNA, and proteins were specified more accurately using BCA feedback procedures. Biomolecular component profiles, calibrated to 100 mg/mL for proteins and 20 mg/mL for other biomolecular components, are shown in Figure S4.

BCA toolbox description. The BCA toolbox is a stand-alone software package, which works with a single raw Raman spectrum measured in a cell on the glass-bottom dish. The program consists of three main blocks: The background processing and subtraction, the nonlinear least squares routine, and the graphic user interface. The following input data was included: (i) Measured cellular spectrum, (ii) specification of the cellular region or organelle where that spectrum was measured (nucleus, nucleolus, mitochondrion, endoplasmic reticulum, apparatus Golgi for growing cells, chromosome or cytoplasmic areas for mitotic cells), and (iii) specification of either the live or fixed (formaldehyde, ethanol) state of the cell. The toolbox delivers the following outputs: (i) Background-free Raman spectrum, (ii) residual spectrum for estimation of modelling quality, (iii) weight coefficients for five biomolecular components (proteins, DNA, RNA, lipids, glycogen), and (iv) the ratio of 1665 cm^−1^ to 1440 cm^−1^ lipid peaks as a parameter of the saturation degree of phospholipids. As additional output data, the software generates a file with a spectrum of organellar phospholipids (the result of subtraction of weighted profiles of proteins, DNA, RNA, and glycogen from background free organellar spectrum). A screenshot of the BCA toolbox is shown in the Supporting Materials (Figure S5).

Cell culture. Measurements were performed on HeLa cells grown in conventional glass bottom dishes (Mattek Co., Ashland, MA, USA), in Advanced DMEM (Thermo Fisher Scientific, Waltham, MA, USA) supplemented with 3% fetal calf serum (Thermo Fisher Scientific, Waltham, MA, USA), glutamax (Thermo Fisher Scientific, Waltham, MA, USA), and antibiotic-antimycotic solution (Thermo Fisher Scientific, Waltham, MA, USA) at 37 °C in a humidified atmosphere containing 5% CO_2_. Before Raman spectroscopy measurements, the cells were transferred into optically transparent high glucose DMEM (Thermo Fisher Scientific, Waltham, MA, USA) supplemented with 25 mM of Hepes.

Organelles labeling. Cytoplasmic organelles were labeled by commercial fluorescent reporters to enable targeted acquisition of Raman spectra in these cellular compartments. The mitochondria were labeled with MitoTrecker Green FM (Thermo Fisher Scientific, Waltham, MA, USA), endoplasmic reticulum (ER) with ER-Tracker Green (ThermoFisher Scientific, Waltham, MA, USA), and Apparatus Golgi with NBD C6 ceramide-BSA (Thermo Fisher Scientific, Waltham, MA, USA), in accordance with the manufacturer’s instructions. The organelle stains generated a weak additional Raman signal in cells, which was then taken into account by the software procedure for background subtraction.

Measurement parameters and BCA accuracy. Each Raman spectrum was acquired for 20 s and accumulated in a series of six subsequent acqusitions. Thus, total integration time for each spectrum was 120 s. This time was found to be sufficient to optimize the signal/noise ratio, and to enable a high quality of BCA, while keeping the unwanted phototoxicity low. Confocality of measurements was enabled by using the aperture device option “50 μm pinhole”. Before the first measurement, the dish was kept untouched for at least 5 min to ensure thermal and mechanical stabilization. This step is critical to avoid movement of the glass bottom during the measurements. The quality of the model fit for organellar BCA was estimated by comparing the residual intensities over the significant wavelength range, with standard error produced by the Raman microscope during measurement of egg white protein mixture (±3 counts per second, cps). If residual intensities were larger than this value, the resulting measurement was rejected.

## 3. Results and Discussion

### 3.1. Raman Spectroscopy and BCA Toolbox Software

In this study, we intended to develop a versatile technology, which could be easily applied to different laboratory settings. We therefore did not use any specialized substrates, such as CaF_2_, which produce less Raman background than glass, but are significantly more expensive. Instead, cells were grown in conventional glass-bottom dishes. After testing several types of cell culture media, we found that cells maintained maximum viability in optically transparent DMEM containing high glucose. We used DMEM for all live imaging experiments. 

As described in the Methods, a precise BCA analysis requires adjustments depending on the type of organelle, as well as whether the cell was live or fixed, and in the latter case, the type of fixative. We have developed a graphic interface to pre-select these variable inputs (Figure S5) and to facilitate data analysis. To establish Raman spectroscopic protocols for fixed cell studies, we have also analyzed cells fixed in either formaldehyde or ethanol.

### 3.2. Results of BCA in Cellular Organelles

To validate the BCA toolbox implemented in the DXR Raman microscope, Raman spectra of different organelles in HeLa cells were acquired, and then the *BCAbox* for the measured spectra was applied. Raman spectra of nuclei, nucleoli, mitochondria, endoplasmic reticulum, apparatus Golgi in growing cells, and chromosomes or cytoplasmic areas in mitotic cells, were measured and analyzed. To estimate the effect of aldehyde and alcohol fixatives on the spectra of the organelles, measurements were performed both in live cells and fixed cells. Examples of Raman raw spectra and preprocessed spectra are shown in the Supporting Materials (Figure S6).

**Figure 1:**
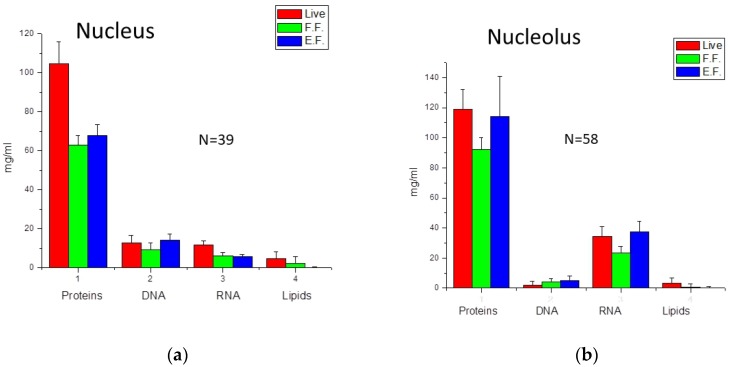

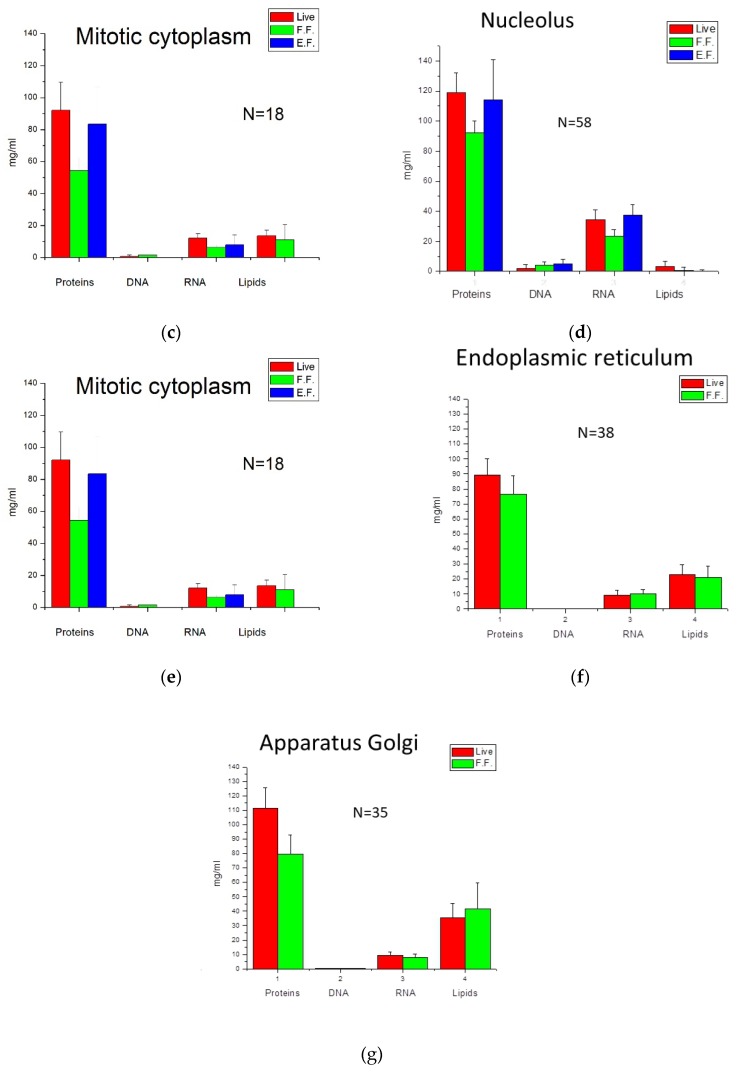
Concentrations of Proteins, DNA, and RNA obtained in different cellular organelles—nuclei (**a**), nucleoli (**b**), mitotic cytoplasm (**c**), mitotic chromosomes (**d**), mitochondria (**e**), endoplasmic reticulum (**f**) and apparatus Golgi (**g**)—in live and fixed cells. Abbreviations: E.F.—fixed by ethanol, F.F.—fixed by formaldehyde. N shows the number of measured organelles. Error bars show cell-to-cell variations of concentrations.

Graphical information on the average values of biomolecular concentrations in organelles and cell-to-cell deviations are shown in Figure 1, and Table 1 and Table 2.

In general, the obtained results were consistent with our previous studies using BCA with other microspectrometers [14]. Each organelle contains a unique set of biomolecular components different from the other. Concentration variations from cell to cell for each organelle on average does not exceed 30%, except for phospholipids in cytoplasm, which can vary up to 80%.

Specific details for each biomolecular component found in the organelles by BCA are discussed below.

**Figure 2:**
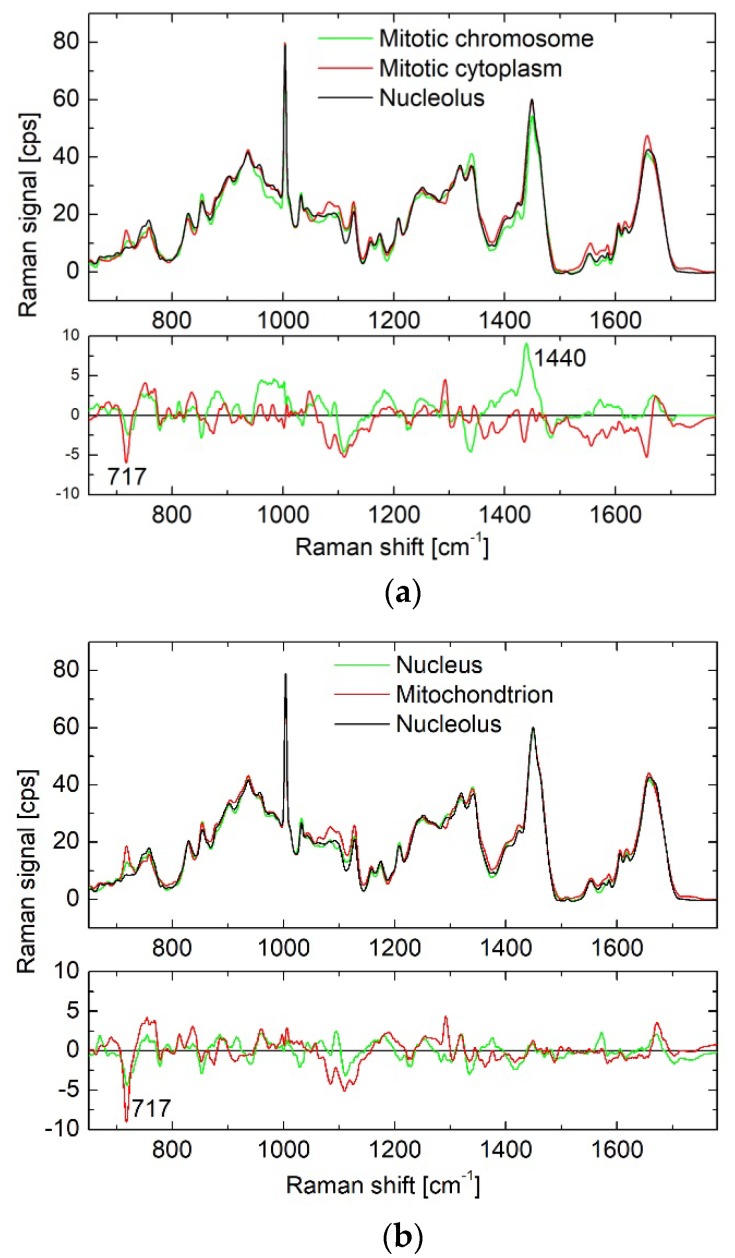

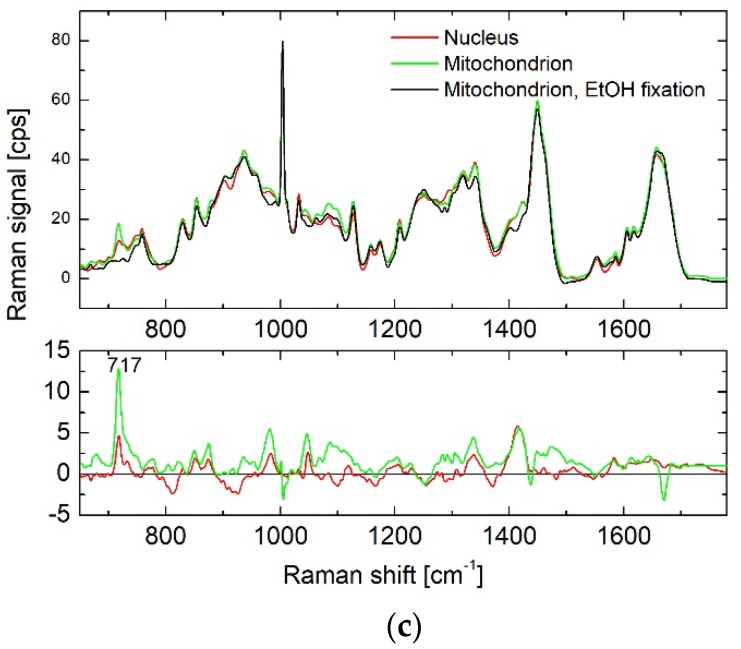
Raman profiles of the protein component - for different organelles (upper curves) and difference spectra (lower curves), obtained by subtraction of the nucleolar profile from that of other organelles, shown in upper part of the graph. Proteins components of nucleoli, mitotic cytoplasm and chromosomes for live cells are shown in (**a**), proteins components of nucleoli, nuclei and mitochondria for live cells are shown in (**b**), and proteins components of nuclei and in live cells and mitochondria in ethanol-fixed cells are shown in (**c**).

Proteins. The protein Raman profiles for all organelles are quite similar (Figure 2). At the same time, intensities in spectra could differ in some specific wavelength ranges, which when the wrong protein component was used for BCA, can result in high intensities of residual spectra. Most prominent differences were found in the ranges of 1200–1300 cm^−1^, 1410–1450 cm^−1^, 1650–1680 cm^−1^, and in the presence of the peak at 717 cm^−1^. Next, we found that the concentration of proteins can decrease by 20–40% in different organelles after fixation in formaldehyde. This decrease is beyond the general heterogeneity variations, and is consistent with previous reports on membrane deterioration and leaking of smaller proteins at the beginning of formaldehyde fixation [10]. The same decrease in protein concentration was observed in nuclei and mitotic chromosomes after ethanol fixation. At the same time, fixation in formaldehyde does not visibly influence the Raman profile of proteins, while ethanol fixation can produce considerable changes in the shape of the spectra, which was discussed in our previous study [10].

As follows from Figure 2, we formally assigned the Raman peak at 717 cm^−1^ to the spectra of cytoplasmic proteins. This assignment is based on the fact of the highest correlation of the peak intensity with the concentration of proteins (Figure S7). At the same time, we do not exclude that this peak can be attributed to phosphatidylcholine [25], which can reach up to 44% of cytoplasmic (mitochondrion) phospholipids [26].

DNA. According to our studies, the DNA component is the most stable biomolecule in the cell nucleus and mitochondria. We did not observe significant changes in the DNA concentration after cell fixation. At the same time, the profile of the DNA component fits well with organellar models during BCA procedures for all organelles and fixation methods.

**Figure 3:**
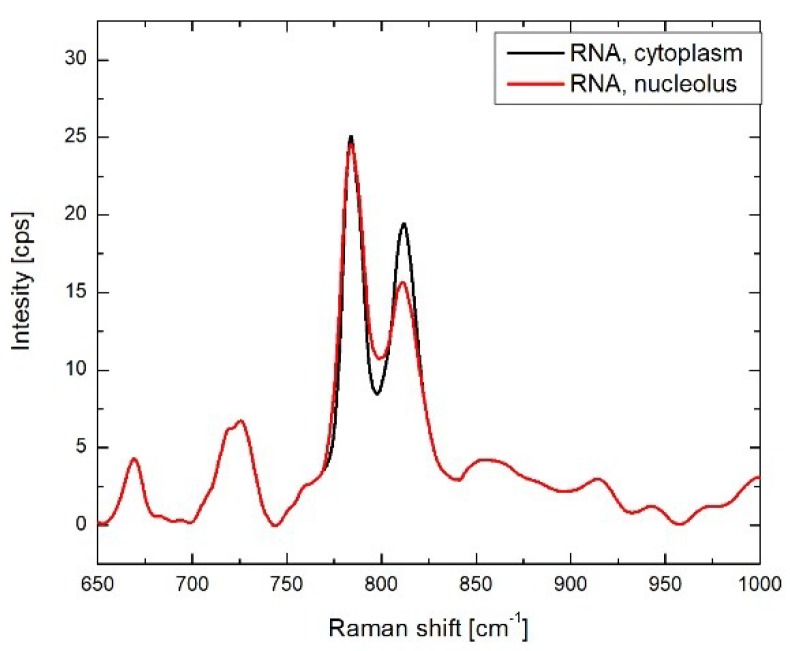
Raman spectra of cytoplasm (black) and nucleolar (red) RNA in the 650–1000 cm^−1^ wavenumber range used in *BCAbox*.

RNA. RNA concentration significantly drops after formaldehyde fixation, except in cytoplasmic organelles. These changes could be potentially attributed to degradation of RNA in the formaldehyde [10]. At the same time, we found that for nuclear compartments and cytoplasmic organelles, two different Raman profiles should be used for the BCA modeling procedure (Figure 3). In cytoplasmic organelles, the peak of RNA at 815 cm^−1^ (O–P–O symmetric stretching) is more intense, which suggests variations in higher order structure for cytoplasmic RNA [27].

**Figure 4:**
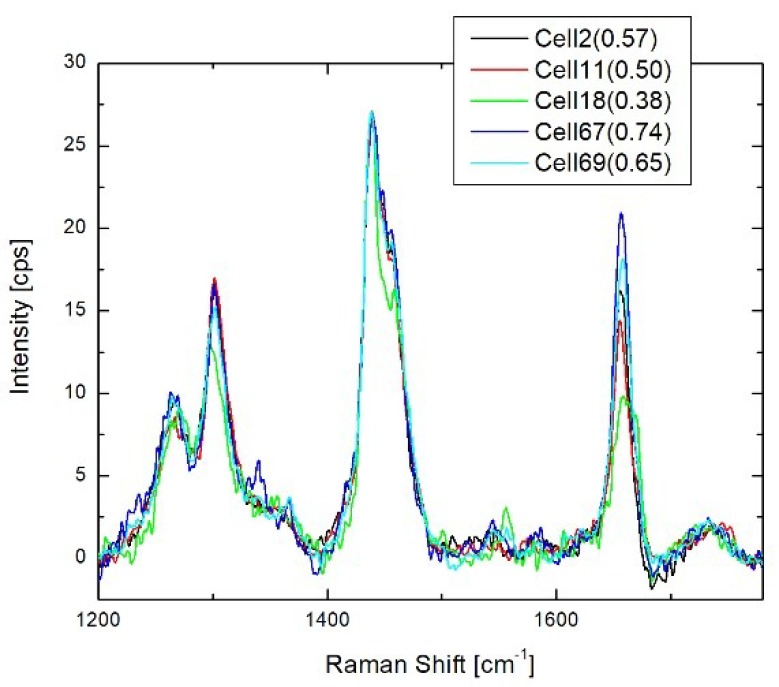
Spectra of endoplasmic reticulum lipids in the range 1200–1800 cm^−1^ normalized to 1665 cm^−1^ peak intensity. Spectra represent different live cells growing in the same dish. Numbers in parentheses show the ratio of peaks at 1665 and 1440 cm^−1^.

Lipids. Biomolecular component analysis indicates a significant diversity of cellular phospholipids. This diversity is mostly determined by the degree of lipid unsaturation, which can be estimated by ratio intensities of Raman peaks at 1665 and 1440 cm^−1^ [25,28]. As an example, Figure 4 demonstrates the lipid spectra of endoplasmic reticulum normalized to the peak at 1665 cm^−1^ for five different cells, where the saturation parameter varies from 0.35 to 0.71. Similar variations were observed for lipid spectra in different cytoplasmic locations of the same cell. Compared to the ratio of 0.58 for monounsaturated oleic acid, our data shows that cytoplasmic phospholipids in cells can contain different fractions of saturated, as well as unsaturated lipids.

## 4. Conclusions

The *BCAbox* software was developed and implemented in the Thermo Scientific DXR Raman confocal microscope. We demonstrated that this software tool is useful for immediate analysis of molecular content in major organelles, including nucleoli, endoplasmic reticulum, Golgi apparatus, and mitochondria of either live or fixed cells. This software package is suitable not only for studies of macromolecular heterogeneity of cell cultures, but can also be applied for detailed analysis of Raman bands of biomolecular components in single organelles. In this study, *BCAbox* was tuned for particular hardware: A DXR2 Raman microscope with a red (633 nm) laser unit of 60 mW output power, a high resolution grating unit, and a high aperture oil immersion objective lens. Besides, it requires careful intensity calibration and can involve additional tasks for sample preparation (e.g., fixation and specific organelles staining). At the same time, the proposed software can be modified for a broad variety of commercially available Raman microscopes, and serve for further advancement and standardization of Raman analysis in biomedical applications. Modification of *BCAbox* for incorporation into Raman imaging software can be the next step to expand the features of Raman microscopy.

## Figures and Tables

**Table 1:** Biomolecular concentrations of Proteins, DNA, and RNA in different cellular organelles (M—mitochondrion, ER—endoplasmic reticulum, AG—apparatus Golgi, M.Cyt.—mitotic cytoplasm, M.Ch.—mitotic chromosome; F.F.—formaldehyde fixation, E.F.—ethanol fixation). Units: mg/mL.

		Proteins			DNA			RNA		
	# of Cells	Live	F.F.	E.F.	Live	F.F.	E.F.	Live	F.F.	E.F.
Nucleus	39	104.6 ± 11.23	63.0 ± 4.9	67.9 ± 5.5	12.5 ± 4.0	9.3 ± 3.2	13.9 ± 3.	11.6 ± 2.0	6.0 ± 1.9	5.6 ± 1.2
Nucleolus	58	119.1 ± 13.2	92.3 ± 8.0	114.0 ± 26.8	1.9 ± 2.5	4.1 ± 2.4	4.8 ± 3.2	34.4 ± 6.5	23.4 ± 4.4	37.2 ± 7.3
M	38	100.0 ± 15.5	76.3 ± 10.1	91.9 ± 12.5	7.4 ± 3.2	6.5 ± 3.2	10.7 ± 7.6	8.5 ± 2.4	7.2 ± 2.4	5.7 ± 1.0
ER	38	89.1 ± 11.2	76.5 ± 12.0	-	0.2 ± 0.1	0.3 ± 0.1	-	9.1 ± 3.3	10.0 ± 3.1	-
AG	35	111.5 ± 14.0	79.6 ± 13.1	-	0.2 ± 0.1	0.3 ± 0.2	-	9.3 ± 2.2	7.7 ± 2.5	-
M.Cyt. ^1^	16	92.1 ± 17.5	54.3 ± 8.3	83.4 ± 23.1	0.7 ± 0.7	1.7 ± 1.6	0	12.2 ± 2.76	6.3 ± 1.7	7.9 ± 6.3
M.Ch. ^2^	18	98.1 ± 14.4	51.9 ± 9.0	67.3 ± 22.1	20.8 ± 8.5	19.7 ± 3.9	30.5 ± 10.5	14.6 ± 2.9	5.0 ± 1.9	8.5 ± 3.9

^1^ Cytoplasmic area of mitotic cell. ^2^ Chromosome area of mitotic cell.

**Table 2:** Concentrations of lipids in different cellular organelles (M—mitochondrion, ER—endoplasmic reticulum, AG—apparatus Golgi, M.Cyt.—mitotic cytoplasm, M.Ch.—mitotic chromosome; F.F.—formaldehyde fixation, E.F.—ethanol fixation). Units: mg/mL.

		Live		F.F.		E.F.	
	# of Cells	Concentration	1665/1440	Concentration	1665/1440	Concentration	1665/1440
Nucleus	39	4.5 ± 3.5	0.62 ± 0.11	2.0 ± 3.5	-	0.1 ± 0.4	-
Nucleolus	58	3.1 ± 3.6	0.65 ± 0.10	0.6 ± 2.1	-	0.1 ± 0.9	-
M	38	15.5 ± 12.1	0.57 ± 0.10	16.7 ± 6.5	0.58 ± 0.10	7.0 ± 10.0	0.63 ± 0.10
ER	38	23.1 ± 6.5	0.54 ± 0.06	21.2 ± 7.3	0.55 ± 0.07	-	-
AG	35	35.4 ± 10.2	0.43 ± 0.05	41.5 ± 18.2	0.49 ± 0.05	-	-
M.Cyt.	16	13.6 ± 3.5	0.53 ± 0.10	11.1 ± 9.5	0.51 ± 0.10	0	-
M.Ch.	18	6.2 ± 1.4	0.52 ± 0.10	4.3 ± 2.7	0.46 ± 0.06	3.0 ± 3.0	0.74 ± 0.06

## Supplementary Materials

The following are available online at http://www.mdpi.com/2079-6374/8/4/106/s1, Figure S1: Raw Raman spectra of egg white and corresponding Standard Deviations; Figure S2: Diagram of the background subtraction; Figure S3: Components of background used in BCA toolbox; Figure S4: Biomolecular component profiles; Figure S5: Screenshot of BCA toolbox; Figure S6: Measured and preprocessed spectra of Apparatus Golgi in HeLA cells; Figure S7: Cytoplasmic proteins and biomolecular concentration correlation analysis.
