# Supplementary material for: BCAbox Algorithm Expands Capabilities of Raman Microscope for Single Organelles Assessment
Source: Biosensors (Basel). 2018 Nov 10;8(4):106. doi: 10.3390/bios8040106 (PMC6316203; doi:10.3390/bios8040106)
Supplement: Supplementary file 1 [file biosensors-08-00106-s001.pdf]

Supplementary Materials

BCAbox algorithm expands capabilities of Raman microscope for single organelles assessment

Andrey Kuzmin[1,2,*], Art Pliss[1,2], Alex Rzhevkii[3], Adrian Lita[4], and Mioara Larion[4]

[1] Advanced Cytometry Instrumentation Systems, LLC, 19 Elm Street, Buffalo, NY 14203; lptacis@gmail.com

[2] Institute for Lasers, Photonics and Biophotonics, University at Buffalo, State University of New York, Buffalo, NY 14260; ampliss@buffalo.edu

[3] Thermo Fisher Scientific, 2 Radcliff Rd., Tewksbury, MA 01876; alexander.rzhevskii@thermofisher.com

[4] Neuro-Oncology Branch, Center for Cancer Research, National Cancer Institute, Bethesda, MD 20892; mioara.larion@nih.gov

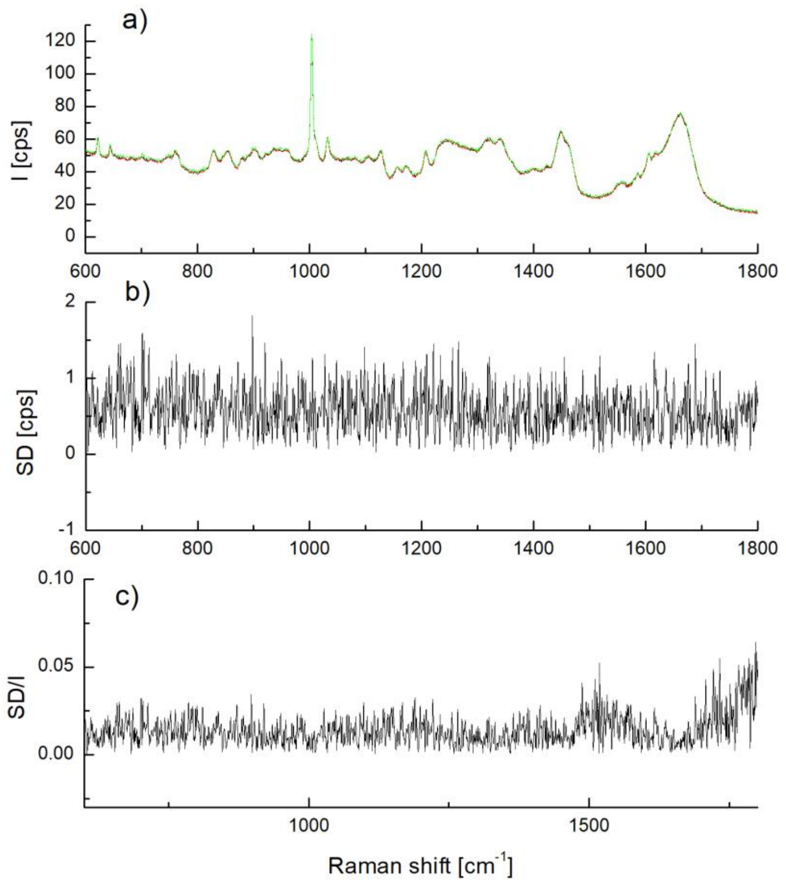

Fig. S1. (a) Three raw Raman spectra of freshly extracted white egg (acquisition 6x20 s). (b) Standard deviations, calculated from (a). (c) The ratio of standard deviations to intensity of one of the spectra from (a).

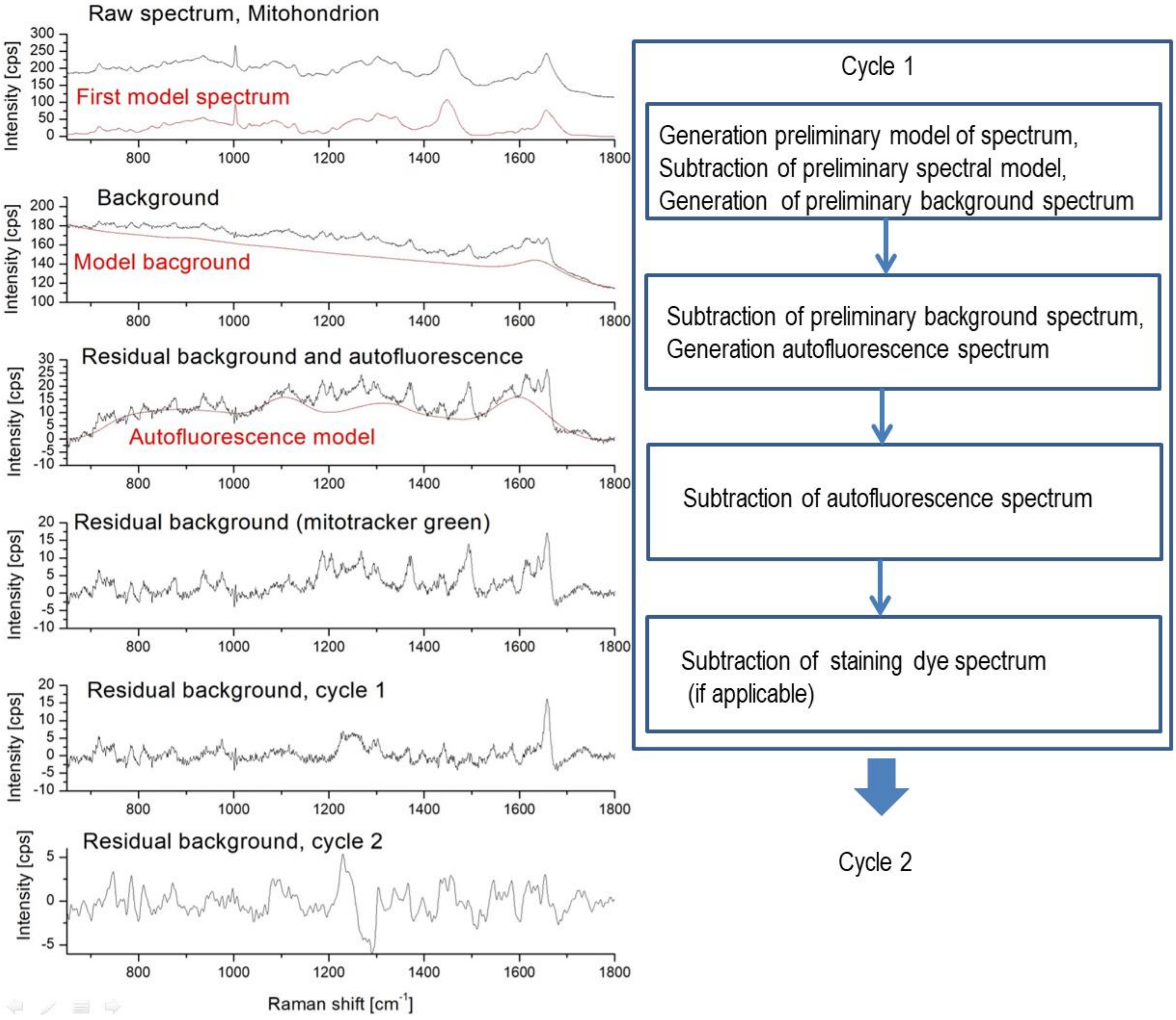

Fig. S2. Simplified diagram of the software algorithm for the background subtraction (right panel) and representative sample of background subtraction for HeLa mitochondrion spectrum (left panel).

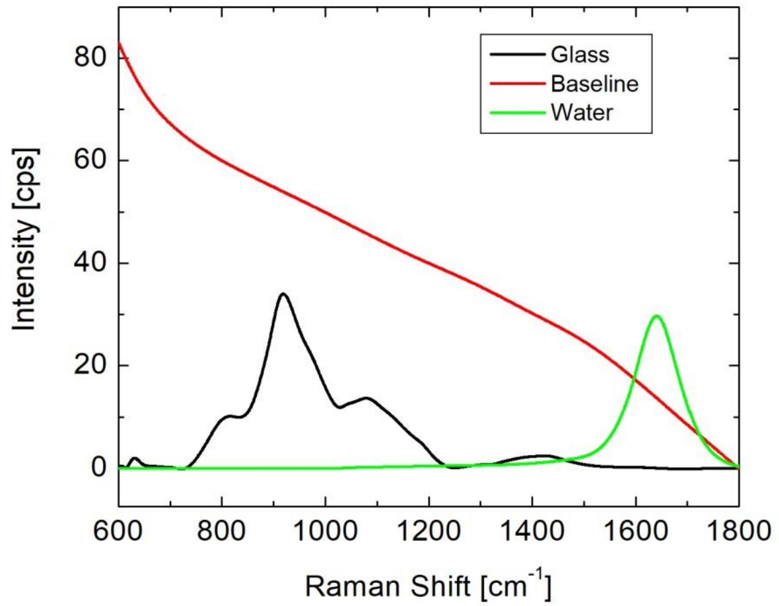

Fig. S3. Spectral components of background used in BCA toolbox, as indicated.

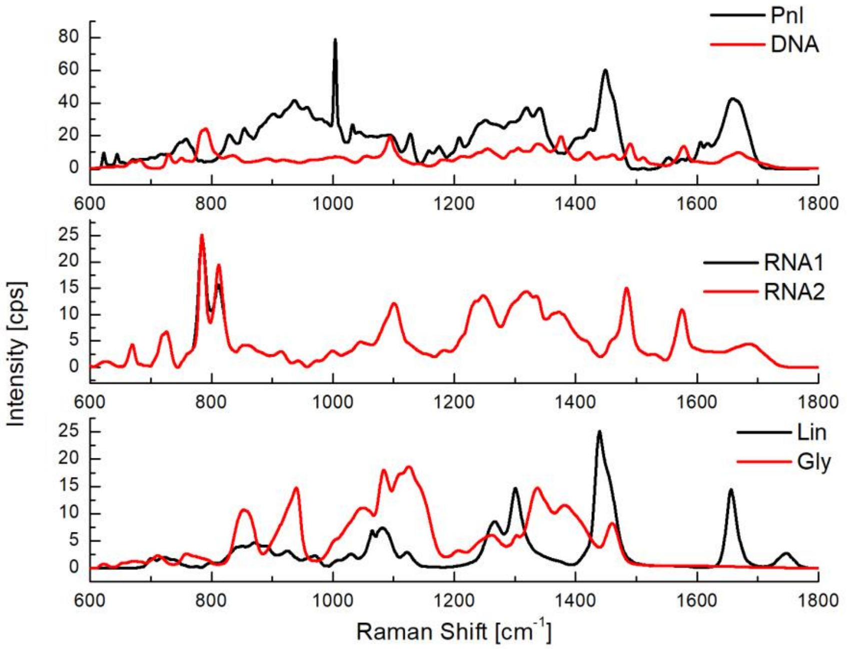

Fig. S4. Biomolecular component profiles. Pnl – nucleolar proteins, RNA1 – nucleolar RNA, RNA2 – cytoplasmic RNA, Lin – phospholipids, Gly - Glycogen. Lin component is used in initial cycle of model fitting; for further cycles the phospholipid profile with specific unsaturation degree, calculated from residual lipid spectrum, is used.

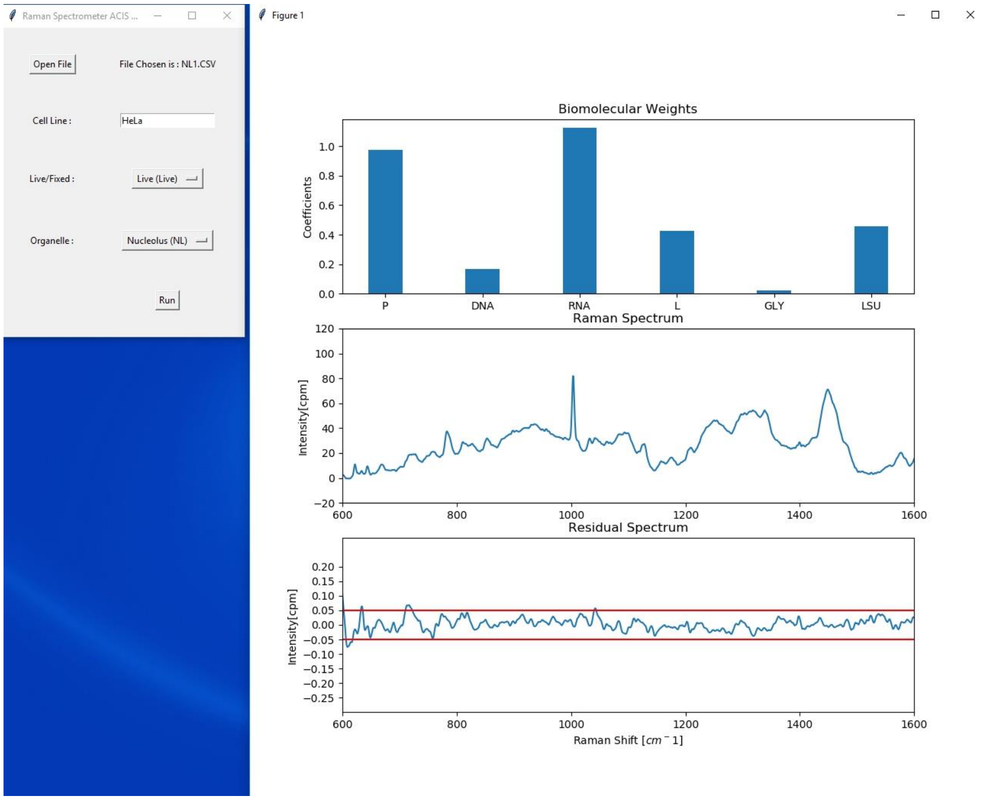

Fig. S5. Screenshot of *BCAbox*. Left panel shows input menu. Right panel contains graphical results of the spectrum analysis.

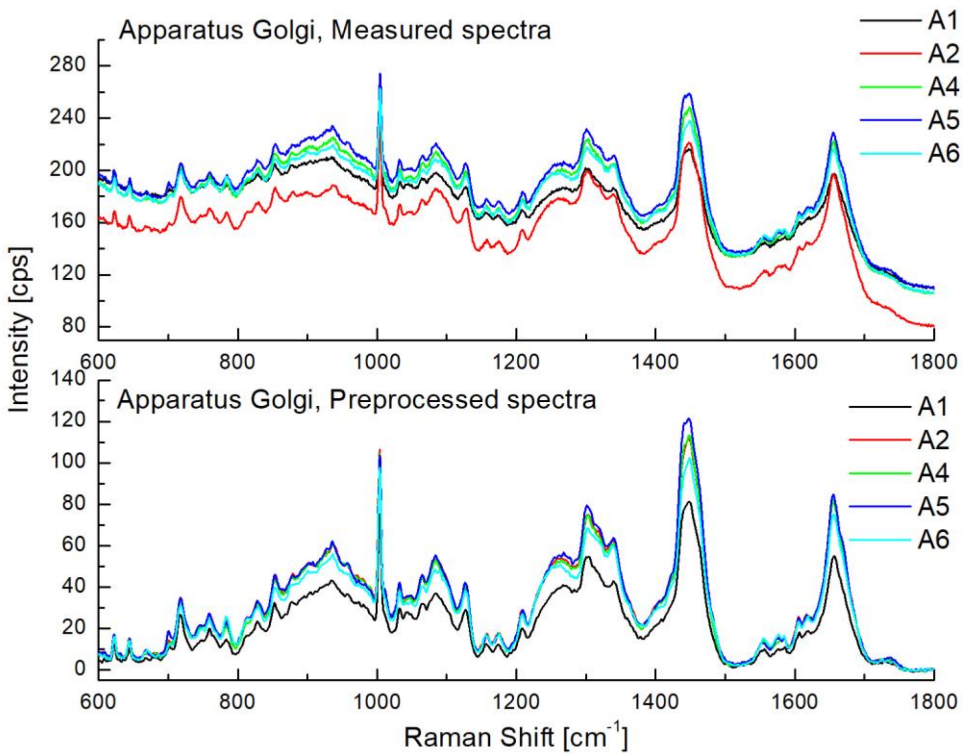

Fig. S6. Measured and preprocessed in *BCAbox* spectra of Apparatus Golgi. Data set contains results from five HeLA cells.

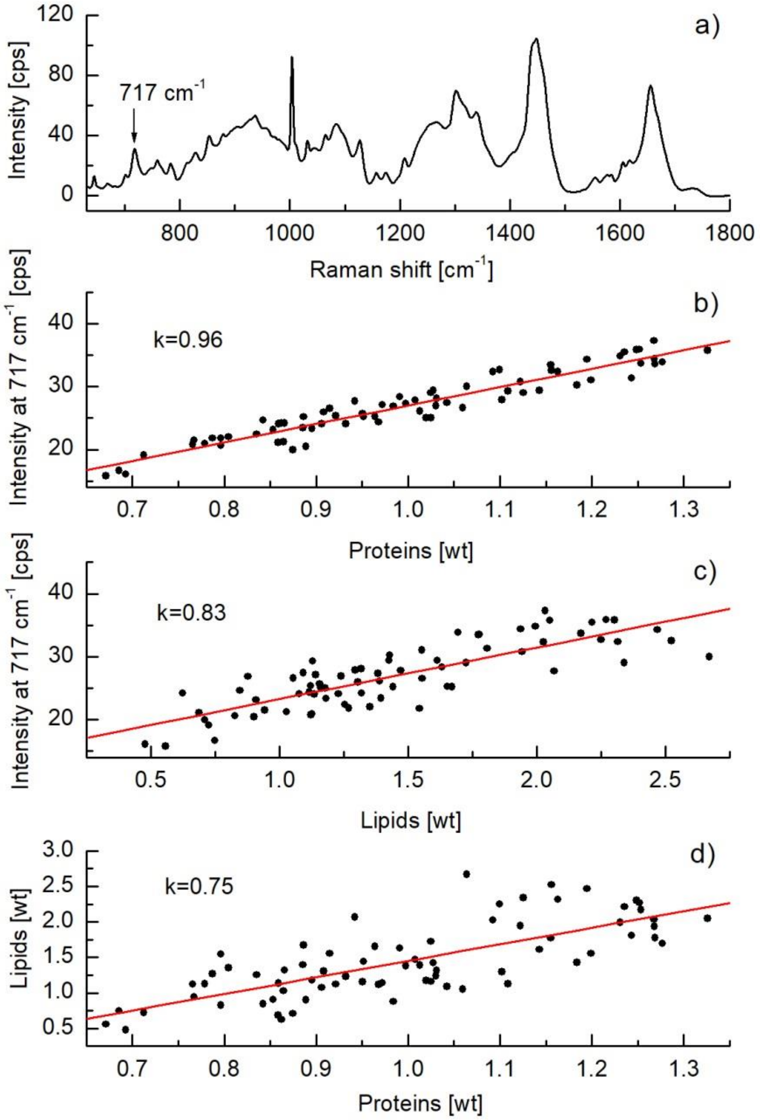

Fig. S7. Cytoplasmic proteins (a), correlation analysis of 717 cm^{-1} peak intensity vs protein's (b) and lipid's concentration (c) and concentration correlation analysis proteins vs lipids (d); k is Pearson coefficient.
